# Feeding Problems in Patients with Noonan Syndrome: A Narrative Review

**DOI:** 10.3390/jcm11030754

**Published:** 2022-01-30

**Authors:** Dagmar K. Tiemens, Leenke van Haaften, Erika Leenders, Annemiek M. J. van Wegberg, Bregtje Gunther Moor, Joyce Geelen, Jos M. T. Draaisma

**Affiliations:** 1 Department of Pediatrics, Radboud Institute for Health Sciences, Radboud University Medical Center, Amalia Children’s Hospital, 6500 HB Nijmegen, The Netherlands; dagmar.tiemens@radboudumc.nl (D.K.T.); joyce.geelen@radboudumc.nl (J.G.); 2 Dutch Noonan Syndrome Foundation, Stationsweg 6b, 3862 CG Nijkerk, The Netherlands; 3 Department of Rehabilitation, Donders Institute for Brain, Cognition and Behavior, Radboud University Medical Center, 6500 HB Nijmegen, The Netherlands; leenke.vanhaaften@radboudumc.nl; 4 Department of Human Genetics, Radboud University Medical Center, 6500 HB Nijmegen, The Netherlands; erika.leenders@radboudumc.nl; 5 Department of Gastroenterology and Hepatology-Dietetics, Radboud University Medical Center, 6500 HB Nijmegen, The Netherlands; annemiek.vanwegberg@radboudumc.nl; 6 Department of Medical Psychology, Radboud Institute for Health Sciences, Radboud University Medical Center, Amalia Children’s Hospital, 6500 HB Nijmegen, The Netherlands; Bregtje.GuntherMoor@radboudumc.nl

**Keywords:** Noonan syndrome, Noonan syndrome spectrum disorder, pediatric feeding disorder, gastroesophageal reflux disorder, increased energy demand

## Abstract

Noonan syndrome (NS) belongs to the group of Noonan syndrome spectrum disorders (NSSD), which is a group of phenotypically related conditions. Feeding problems are often present not only in infancy but also in childhood, and even beyond that period. We describe the different aspects of feeding problems using a (theoretical) concept proposed in 2019. More than 50% of infants with NS develop feeding problems, and up to half of these infants will be tube-dependent for some time. Although, in general, there is a major improvement between the age of 1 and 2 years, with only a minority still having feeding problems after the age of 2 years, as long as the feeding problems continue, the impact on the quality of life of both NS infants and their caregivers may be significant. Feeding problems in general improve faster in children with a pathogenic *PTPN11* or *SOS1* variant. The mechanism of the feeding problems is complex, and may be due to medical causes (gastroesophageal reflux disease and delayed gastric emptying, cardiac disease and infections), feeding-skill dysfunction, nutritional dysfunction with increased energy demand, or primary or secondary psychosocial dysfunction. Many of the underlying mechanisms are still unknown. The treatment of the feeding problems may be a medical challenge, especially when the feeding problems are accompanied by feeding-skill dysfunction and psychosocial dysfunction. This warrants a multidisciplinary intervention including psychology, nutrition, medicine, speech language pathology and occupational therapy.

## 1. Introduction

Noonan syndrome (NS) belongs to the group of Noonan syndrome spectrum disorders (NSSD), which is a group of phenotypically related conditions. These syndromes are caused by germline pathogenic variants in genes within the Ras/mitogen-activated protein kinase (Ras/MAPK) signalling pathway [1]. The most prevalent syndrome is NS (OMIM 163950). The clinical presentation is extremely variable. Other NSSDs are Noonan-like syndrome with loose anagen hair (NS-LAH; OMM 607721), Noonan syndrome with multiple lentigines (NSML; OMIM 151100), Noonan syndrome-like disorder (CBL; OMIM 613563), Costello syndrome (CS; OMIM 218040) and cardiofaciocutaneous syndrome (CFCS; OMIM 115,150) [1]. Growth problems in NS have received much attention, as can be illustrated by the fact that growth was one of the criteria in the scoring system of van der Burgt [2]. However, several aspects of feeding problems and energy expenditure, which may influence growth and well-being, have received less attention. For this reason, this narrative review will focus on these topics. For several reasons, we have consciously chosen to describe the literature on the feeding problems of the total group of patients with NSSD, instead of only NS in this article. This is not only because of the phenotypical similarity but also because of the fact that some genes may be responsible for more than one NSDD [1]. Moreover, several interesting articles include patients with a different NSSD without making a clear distinction between them, as was the case in the publications on this topic published before the first causing gene (*PTPN11*) was found in 2001 [3]. However, whenever possible we will primarily review the literature on NS and our own experiences. This article aims to help to unravel the complex situation of feeding problems (which may in itself constitute a complex problem) and energy requirements, due to the disease itself or comorbidity. In order to achieve this goal, it is important to have a good definition of feeding problems, which will be discussed first.

We will also underline the possible impact of this complex situation on children and caregivers. Moreover, we will discuss the literature and our own experiences of these problems in adolescents and adults with NS.

## 2. Definition of Feeding Problems

As was postulated before, feeding problems in themselves may constitute a complex problem. However, in order to unravel the complexity we will first discuss two recent definitions of feeding problems.

The first definition, a (theoretical) concept, was proposed in 2019; it is called Pediatric Feeding Disorder: impaired oral intake that is associated with medical, nutritional, feeding skill, and/or psychosocial dysfunction, and is not age-appropriate [4]. This concept includes (aspects of) dysphagia and Avoidant/Restrictive Food Intake Disorder (ARFID). Moreover, this concept may also be used in adolescents and adults. The essential diagnostic criteria are:

A: Impaired oral intake that is not age-appropriate, lasting at least 2 weeks and associated with one (or more) of the following types of dysfunction:

- Medical dysfunction: Cardiorespiratory dysfunction, aspiration or motility and functional gastrointestinal disease.
- Feeding-skill dysfunction: The need for texture modification, the use of modified feeding position, or the use of modified feeding strategies.
- Nutritional dysfunction: Malnutrition, the restricted intake of one or more nutrients, or reliance on enteral feeds or nutrients supplements.
- Psychosocial dysfunction: Active or passive avoidance behaviors (including those due to post-traumatic stress disorder) and the disruption of social functioning or the caregiver–child relationship within the context of feeding.

B: The absence of cognitive processes which are consistent with body image disturbances.

This definition also includes (aspects of) the commonly used terms “dysphagia” and “Avoidant/Restrictive Food Intake Disorder (ARFID)”. Dysphagia can be defined as impaired oral, pharyngeal and/or esophageal phases of swallowing. Dysphagia can occur in association with gastro-esophageal reflux disease [5]. Due to the dysphagia, many children develop adaptive feeding behaviors. Dysphagia can also be seen as one of the elements of Pediatric Feeding Disorder, namely a skill dysfunction.

ARFID is an eating or feeding disturbance (e.g., an apparent lack of interest in eating or food, avoidance based on the sensory characteristics of food, or concern about the aversive consequences of eating) manifested in the persistent failure to meet appropriate nutritional and/or energy needs. The definition acknowledges that feeding disorders are common in certain medical conditions; however, it requires that the severity of the eating disturbance exceeds that associated with the condition, and specifically excludes children whose primary challenge is a skill deficit [4,6]. ARFID can also be seen as part of the elements of Pediatric Feeding Disorder, namely psychosocial dysfunction.

The second definition is a functional approach which was also presented in 2019 [7]. This approach uses “red flags” for which additional investigation and interdisciplinary intervention is required [7,8]. These “red flags” include dysphagia, aspiration, vomiting, growth failure, force feeding and developmental delay, symptoms which are often present in children with NSSD [9].

For this article, we decided to use the definition of Pediatric Feeding Disorder, due to its clear definition. However, before discussing the different modes of dysfunction, we will start by describing the prevalence of feeding problems and their natural history.

## 3. Prevalence of Feeding Problems

Feeding problems in children with NSDD were reported long before the genetic diagnosis of NS could be performed. Feeding problems may present as an absence of interest, poor sucking, prolonged feeding times and recurrent vomiting [10]. In this paragraph, no distinction is made in the type of dysfunction.

Already, in 1992, a study was published which stated that more than 75% of 151 patients clinically diagnosed with NS (aged one week to 60 years old (mean: 12.6 years)), experienced feeding problems [10]. Almost one third of these infants required tube-feeding. In another study, published in 1999, 64% of 25 children (median age 3.2 years; range 2 months to 12 years) clinically diagnosed with NS experienced feeding problems [11]. Shaw et al. reported that 65% of 91 infants with clinically diagnosed NS experienced feeding problems in the first months of their lives, of which 36% were tube-dependent [12]. Furthermore, the prevalence of feeding problems in 134 infants with NSDD was reported to be 55 to 100%, depending on the type of NSDD [9]. Among children with CFCS, a high frequency of gastrointestinal problems was also found, with a prevalence of failure to thrive of 82%, and a prevalence of assisted feeding of 51% [13]. This is in contrast with the prevalence of feeding problems in the general population. Although 25 to 50% of young children were reported to have feeding difficulties, only about 10% of them were severe enough to require intensive intervention [7]. Many symptoms were a consequence of dysmotility, including swallowing difficulties, frequent forceful vomiting, gastroesophageal reflux disease, and failure to thrive. Recently, we described feeding problems in 56% of 52 infants with genetically proven NS [14]. More than half of these infants (59%) were tube-dependent. In a more recent study of 108 children, the prevalence of feeding problems in the first year was 52%, and more than half of these infants were tube-dependent [15].

## 4. Natural History of Feeding Problems

The natural history of feeding problems has seldom been investigated. Shah et al. (1999) mentioned that feeding problems remarkably improved after the age of 3 to 4 years [11]. However, in a more recent article, it was mentioned that the feeding difficulties usually resolve in the first few years of life [16]. In our study of 108 patients, we found a major improvement of the feeding problems between the ages of 1 to 2 years in patients with feeding problems in infancy, with only a minority still having feeding problems after the age of 2 years [15].

However, feeding problems may also develop after the age of one year. Fifteen percent of the children in our study developed feeding problems after the age of one year [15]. Most of these children developed feeding problems in the 1–2 years age group, one child developed them in the 2–4 years age group, and four children developed them in the 4–6 years age group. Nine percent of children with a pathogenic *PTPN11* variant developed late-onset feeding problems; this was after an intercurrent infection, post-operatively and due to psychosocial dysfunction. Almost 20% of the children with a pathogenic *SOS1* variant developed late-onset feeding problems due to delayed gastric emptying, an eating aversion caused by psychosocial dysfunction, or heart disease. Seven out of the group of 25 children (28%) with other pathogenic gene variants developed late-onset feeding problems mostly not directly related to the causes of the early-onset feeding problems. In only one patient were the feeding problems primarily caused by psychosocial dysfunction. Of the children with other pathogenic gene variants, more than 50% were tube-dependent for some time.

There is less literature on feeding problems in adolescents and adults. In a study on the nutritional aspects of Noonan syndrome and Noonan-related disorders, which included 62 individuals aged from 2 to 56 years, only one patient with CFCS reported feeding difficulty and required a gastrointestinal tube and gastrostomy. Sporadic episodes of nausea and vomiting were reported by four individuals, and were ameliorated without treatment [17].

In the study of Smpokou et al. of 35 patients with NS aged 18–68 years old, 60% of the patients experienced complaints of gastroesophageal reflux disease, which started between the age of zero and 44 years of age [18].

Our own experience is that we seldom see adolescents or adults with a NSSD with the need for tube-feeding. However, we encounter adolescents and adults with oral diet with dietary modifications, for instance due to problems with the structure of food, or complaints of gastroesophageal reflux disease.

## 5. Genotype–Phenotype Correlation in Noonan Syndrome

In our study, children with a pathogenic gene variant other than *PTPN11* and *SOS1* had significantly more feeding problems in the first year [15]. In Table 1, the prevalence of feeding problems of infants with NS due to the most prevalent pathogenic gene variants is given. In our study, 20 of the 63 (32%) infants with a pathologic *PTPN11* variant needed tube feeding, along with 2 of the 16 patients (12.5%) of the infants with a pathologic *SOS1* variant.

Although Digilio et al. [9] described the pathologic variants of *PTPN11*, *SOS1* and *RAF1,* they did not correlate these specific pathologic variants with feeding disorders.

In children with NS due to pathogenic gene variants other than *PTPN11* or *SOS1* (for example, pathogenic variants of *KRAS*, *RAF1*, *RIT1* and *SOS2*) or other NSSDs (NS-LAH, NSML, CFCS, CBL, CS), feeding problems persisted significantly longer than in patients with a pathogenic *PTPN11* or *SOS1* variant. Two of the 19 children with NS or other pathogenic gene variants than *PTPN11* or *SOS1,* or with other NSSD, were tube-dependent at the age of 6 years. However, only one out of 63 NS patients with a pathogenic *PTPN11* variant had a modified diet, and none were tube-dependent at the age of 6 years. Furthermore, none of the 16 patients with a pathogenic *SOS1* variant were tube-dependent or had an oral diet with restrictions.

## 6. Medical Dysfunction as a Cause of Feeding Problems

It is not exactly known what medical dysfunction(s) causes the feeding problems. Gastro-oesophageal reflux disease and delayed gastric emptying were documented in 44% of children with feeding problems by Shah et al [11]. We documented gastroesophageal reflux disease as a presumed or proven cause in more than two-thirds of infants with feeding problems [15]. Delayed gastric emptying may exacerbate any tendency to gastroesophageal reflux disease [11]. Gastric emptying studies can be of help in the diagnosis of delayed gastric emptying [19]. From personal experience, we can confirm that delayed gastric emptying may play a role in the feeding problems [15]. Shah et al. postulated that the cause of the feeding problems is related to a delayed gastrointestinal motor development, and thus with delayed gastric emptying and gastro-oesophageal reflux disease, and that, in general, this improves with age [11]. Although this may be true, prompt treatment is of the utmost importance not only to prevent oesophageal damage but also because these negative experiences (pain, nausea, vomiting) may contribute to the feeding problems. This may especially be the case in co-morbidity with infections in this region. The recurrent infection of the (middle) ear and/or throat may add to the negative experiences in the otolaryngeal region, and may contribute to feeding problems. In some cases, each problem in itself—and also in co-morbidity—may lead to the development of psychosocial dysfunction (ARFID) [20]. The optimal treatment of gastroesophageal reflux disease and delayed gastric emptying in patients with NS is not yet known. Joint recommendations for the treatment of gastroesophageal reflux disease with or without delayed gastric emptying in general are given by the North American Society for Pediatric Gastroenterology, Hepatology and Nutrition (NASPGHAN), and the European Society for Pediatric Gastroenterology, Hepatology and Nutrition (ESPGHAN) [21]. In these recommendations, the use of ondansetron is not included. We have good experience with the use of ondansetron in children with NS with gastroesophageal reflux disease with or without delayed gastric emptying. Feeding problems in infancy show a correlation with a delay in developmental milestones and special educational needs, and have been suggested to be a marker of poorer long-term outcomes [12]. However, to our best knowledge, this has not been confirmed in other studies.

The onset of feeding problems after the age of one year, in general, is due to postoperative circumstances, infections or co-morbidities. However, infections and, for example, (surgery for) heart diseases are more common in children with NS [14,22]. As such, having NS can be an indirect factor of late-onset feeding problems triggered by, for example, an infection. This was illustrated by a case report which reported that delayed gastric emptying may even present for the first time during adulthood [23]. No child was diagnosed with celiac disease, although Quaio et al. noted that physicians should be alert to the possibility of autoimmune diseases, including celiac disease, in patients with NS [24].

## 7. Feeding-Skill Dysfunction

Feeding-skill dysfunction may present as problems with nippling (breast or bottle), eating from a spoon, drinking from a cup, and biting and chewing [25]. In children with NS, a combination of factors might be the cause of the disturbed development of feeding skills. The dysfunction of eating skills is seen as a result of the complex interactions between anatomical, medical and psychosocial factors, such as in children with other genetic syndromes [26]. Moreover, altered eating experiences in the first year of life due to illness (i.e. gastro-esophageal reflux disease, delayed gastric emptying, infection), or developmental delay may lead to feeding-skill dysfunction.

In the literature, little is known about the precise feeding-skill dysfunction in NS. Only a minority of children with NS require a modified oral diet after the age of 2 years [15]. In clinical practice, we can roughly distinguish between two groups of NS children with feeding-skill dysfunctions. The first group has a delayed development of feeding skills. Because they have less experience, these children show a reduced refinement of oral motor functioning (i.e. inefficient intake, messy eating, and the poor control of liquids, foods and saliva) and, to a lesser extent, they show an impairment in oral sensory functioning. They have problems with the acceptance and tolerance of the liquids and food textures expected for their age [4]. The second group shows a very limited feeding repertoire because a strong, persistent gag reflex hampers the development of oral motor feeding skills. The impairment of oral sensory functioning is the most important factor in this group, and negative experiences—such as from gastroesophageal reflux disease—may contribute to the avoidance of food.

Both groups may benefit from naturally smooth food, modified textures (blending solids) or special feeding equipment or (behavioural) strategies. Treatment approaches such as ‘cue based feeding’ and the principles of motor control and motor learning play an important role in the prevention of feeding skill problems [25,27]. A program that teaches patients to overcome their feeding skill problems must be realistic and based on the development of feeding skills. Besides this, it must be safe, and must support optimal growth and nutritional intake [25]. An individualized, developmentally appropriate treatment may cause less negative experiences (like gagging), and may enhance the development of feeding skills.

## 8. Nutritional Dysfunction

Additionally, it is possible that a need for greater energy intake is initially masked by feeding problems. Both energy supply and energy demand (metabolism, energy expenditure, and the storage of energy in muscle, adipose and bone tissue) participate in the regulation of energy homeostasis [28,29,30]. It is important to evaluate all of the nutritional and feeding aspects involved not only in length growth but also in maintaining a healthy weight for length (BMI) and a healthy body composition.

Several studies have reported on a decrease in BMI with or without reporting on an increased energy demand. A study on eight NS-LAH (pathogenic *SHOC2* variants) patients all showed a failure to thrive, which was also described in a case report [31,32]. Additionally the study of da Silva (2016) also showed a general decrease in BMI in other NSSDs, with the greatest decrease seen in patients with pathologic *SHOC2* and *KRAS* variants [17]. The patients in this study revealed more noticeable effects on the decline of muscle tissue than of adipose tissue. In another study, most of the patients with NSML displayed a lower-than-average BMI, which correlated—for the few patients that could be tested—with reduced adiposity [33].

Malaquias et al. (2012) showed a lower BMI in patients from 7 to 17 years of age with NSSD [34]. They wondered if pathogenic variants in genes associated with NSSD could influence the metabolism and control of energy storage, as two important hormones involved in unsatiety signals (insulin and leptin) also act through the Ras/MAPK pathway. Additionally, the macronutrient distribution in diets compared to a reference population did not show significant differences, indicating that patients with an NSDD need more macronutrients to maintain a BMI that is comparable to a reference population [17]. A study in patients with CS displayed an increased resting energy expenditure and a high calorie intake compared with the recommended levels of energy intake [35]. As such, food intake problems may be even more important because of the growing evidence that a pathogen gene variant in a component of the Ras/MAPK pathway could play a role in a decreased BMI, increased energy expenditure, altered energy storage, and an altered constitution of muscle and adipose tissue.

Although all of these studies indicate that an NSSD results in a higher need for energy intake in order to maintain a healthy BMI, little is known about the exact amount of food or energy intake (e.g., in terms of macronutrient amounts) that these patients may require [32].

## 9. Psychosocial Dysfunction

To our knowledge, there is no direct literature addressing the link between psychosocial mechanisms and feeding problems in young children with NS [15]. However, it is known that feeding problems in infants and young children are mostly multi-factorially determined by issues like medical and/or developmental history, problems with motor control and function, problems with swallowing and behavior, and negative experiences in the oropharyngeal area (e.g., frequent vomiting and/or infections of ears and throat), which may all play a role and may interact with each other [36]. From a psychosocial point of view, two different processes can be distinguished which may contribute to feeding problems in young children. At the child’s level, negative feeding experiences (like gagging, frequent vomiting, pain, or feeling pressured) may result in negative associations with food [37,38]. The child may develop strategies to avoid these feeding situations. When these strategies succeed, the behaviors and the learned feeding avoidance become strengthened [4]. At the level of the caregivers, feeding problems in young children can lead to feelings of insecurity and uncertainty, which may lead to dysfunctional mealtime interactions [39]. This may interfere with a relaxed eating pattern. In other cases, parents may feel or be advised that natural feelings of hunger will eventually resolve the feeding problems, as is the case in hunger provocation [40]. As children with an NSSD may have a higher-than-normal energy expenditure, this strategy may differ from healthy infants, and may contribute to a low BMI. Caregivers may also feel that the feeding problems are too complex to be solved without clinical care, and may accept the ‘status quo’ until intensive multidisciplinary care is available. These examples underline the importance of paying sufficient attention to the caregivers’ feelings and beliefs, and of coping with the feeding difficulties of their infant, as these may play a role in the complex situation of feeding problems.

Feeding difficulties may be treated by cognitive-behavioral interventions which are designed to increase dietary volume and variety [41]. That this may succeed was shown in a meta-analysis of 11 studies evaluating treatment programs for increasing dietary volume in children with ARFID, most of whom had significant co-occurring medical problems; this demonstrated an overall 71% success rate, which was defined by the elimination of dependence on enteral feeds [42]. Most of these treatment programs implemented elements of behavioral therapy. Furthermore, when dysfunctional mealtime interactions developed, these could be treated by analyzing and supporting the parent–child interaction [39]. Importantly, the meta-analysis of Sharp et al., published in 2017, highlighted the benefit of an intensive multidisciplinary treatment, which may take place in day hospital programs and inpatient settings for children with severe feeding difficulties [42]. The core disciplines in such teams include psychology, nutrition, medicine, speech language pathology and occupational therapy.

## 10. Conclusions

Feeding problems appear in more than 50% of infants with NS. About half of these infants are, for a certain time, tube-dependent. In general, there is a major improvement between the age of 1 and 2 years, with only a minority still having feeding problems after the age of 2 years. As long as the feeding problems continue, the impact on the quality of life of both NS infants and their caregivers may be significant. The feeding problems seem to be due to gastro-intestinal motility problems that may persist into adult life. These problems may be accompanied by heart disease, a high rate of infections, and increased energy expenditure. Therefore, treating the feeding problems may be a medical challenge, especially when the feeding problems are accompanied by feeding-skill dysfunction and psychosocial dysfunction. The complexity of impaired oral intake and the impact on the quality of life sometimes require intensive multidisciplinary intervention which includes expertise on psychology, nutrition, medicine, speech language pathology and occupational therapy. Because little is known about the specific treatment of gastroesophageal reflux disease with or without delayed gastric emptying, or about the possible increased energy demand, eating-skill dysfunction and psychosocial dysfunction in relation to feeding in young children with NS, it is important that research on these topics is performed in order to provide more effective treatments.

## Figures and Tables

**Table 1:** Prevalence of feeding problems in the first year of life in patients with NS with the most prevalent gene mutations.

	*PTPN11*	*SOS1*	*RAF1*
Digilio et al., 2012 [9]	13/20 = 65%	0/8 = 0%	3/4 = 75%
Draaisma et al., 2020 [15]	36/63 = 57%	6/16 = 37.5%	2/4 = 50%

## Data Availability

The data presented in this study are openly available from the authors.
